# An acutely erythematous, oedematous penis and antecubital fossae rash in a patient taking etanercept: a case report

**DOI:** 10.1186/1757-1626-2-9116

**Published:** 2009-11-30

**Authors:** Tim Orr, Aidan Noon

**Affiliations:** 1 Department of Urology, Barnsley Hospital, Gawber Road, Barnsley, S75 2EP, UK

## Abstract

**Introduction:**

Acute erythema and oedema of the genitalia is an alarming complaint for any patient. Diagnosis can be complicated by atypical presentation and the use of concurrent immuno-modulatory drugs.

**Case presentation:**

We present a case report of a man on anti-TNF therapy for rheumatoid arthritis presenting with an acutely red, swollen, non-tender penis and scrotum presumed to be infective. The discovery of erythematous plaques in both antecubital fossae alerted the clinicians to consider alternative dermatological diagnoses.

**Conclusion:**

The accepted adjuncts to confirming or excluding infectious aetiology were complicated by the use of immuno-modulatory medication in this case. This patient's unusual presentation may have been associated with and was complicated by the use of etanercept. The case illustrates the need to consider other diagnoses and obtain appropriate advice when the clinical course is not progressing as anticipated.

## Background

An acute erythematous, oedematous penis and scrotum is a frightening complaint for patients, requiring urgent evaluation to rule out a progressive and destructive process [1]. Differential diagnoses include emergencies such as an incarcerated hernia and Fourniere's Gangrene to less immediate problems such as epididymo-orchitis, cellulitis and contact dermatitis [2-4]. Acute idiopathic peno-scrotal oedema has been reported in adults with no history of trauma, allergies or urinary symptoms. Resolution of symptoms has been reported within 72 hours with empirical treatment of scrotal support and elevation, antibiotics and antihistamines [5,6]. We describe an acute presentation of painless, erythematous, swollen genitallia associated with a simultaneous dermatological reaction in both antecubital fossae in a patient taking methotrexate and etanercept for rheumatoid arthritis.

## Case presentation

**Figure 1:**
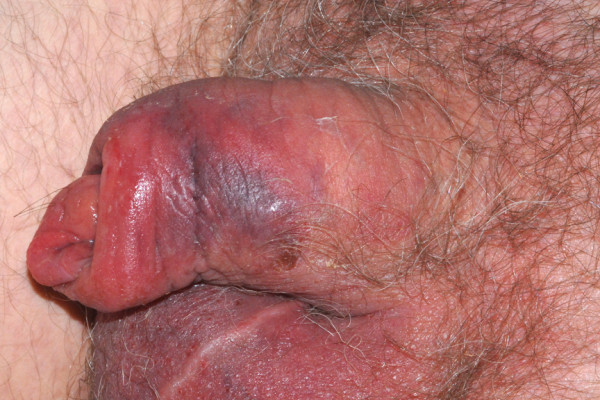
**Image of penis and scrotum**.

A 67-year old white Caucasian man presented to accident and emergency with a 2-day history of an acutely erythematous and oedematous penis and scrotum (see figure 1). Initial investigations revealed a normal full blood count, urea & electrolytes, urinalysis and bacterial swabs. His C-reactive protein was mildly elevated at 16.7 mg/L which was thought to be compatible with his diagnosis of rheumatoid arthritis.

**Figure 2:**
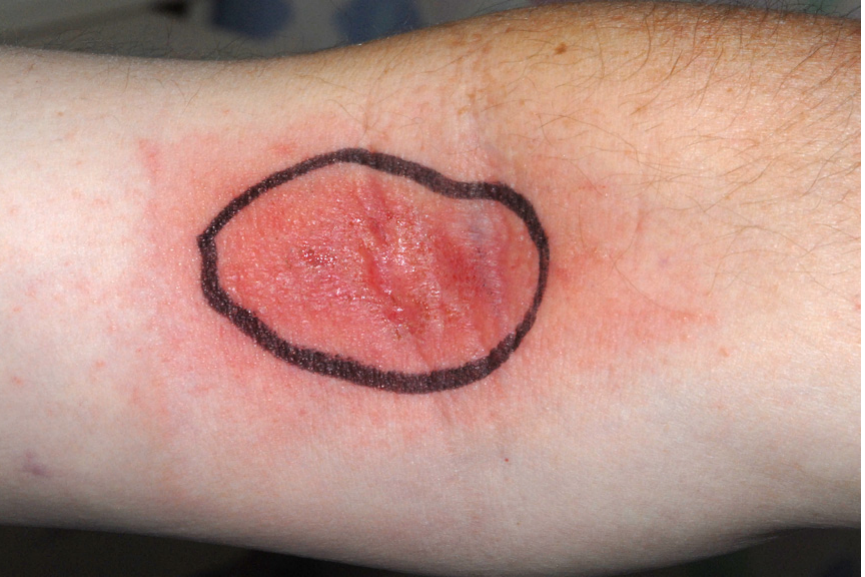
**Image of ante-cubital fossa rash**.

He was commenced on intravenous flucloxacillin and benzylpenicillin. After 48 hours no discernible improvement was noted. He was re-examined and found to have a bilateral pruritic antecubital fossa rash (figure 2) which he reported had developed at the same time as his genital swelling. A full dermatological history was taken and the patient denied any previous rash or localized trauma. Past medical history included atrial fibrillation for which he was on warfarin and rheumatoid arthritis for which he had been on methotrexate for several years and etanercept for 3 months. A dermatological opinion was sought and differential diagnoses were given which included: cellulitis of the penis with a para-infectious skin reaction in his antecubital fossae, an allergic contact dermatitis, or an atypical presentation of psoriasis. The advice of a microbiologist and a rheumatologist was also obtained. He was treated empirically with antibiotics, antihistamines, topical steroids and emollients and gradually improved over the next few weeks.

He returned for skin patch testing which showed reactions to multiple allergens but none which could be implicated in this presentation on careful questioning.

No clear diagnosis was reached at the end of this case. The patient improved on a range of empirical treatment and continues to take methotrexate and etanercept with no further problems to date.

## Discussion

The diagnosis in this case was complicated by an absence of pain, lack of an increase in blood inflammatory markers and leucocytes and the presence of a dermatitis-like skin reaction. The patient failed to improve with antibiotics alone and only after the addition of antihistamines, topical steroids and emollients did his condition start to resolve.

Etanercept is an anti-TNF∝ used in the treatment of a range of inflammatory conditions including rheumatoid arthritis. It is an immuno-modulator and therefore increases the risk of bacterial infections [7,8]. It may also affect the response of acute phase proteins such as CRP and the leukocyte response to infection complicating diagnosis.

Etanercept has also been associated with the development of psoriasis. This has not been extensively studied but it is thought that the alteration in immunity caused by anti-TNF therapy may precipitate psoriasis in pre-disposed individuals [9].

Although allergic contact dermatitis was a differential in this case the distribution of the rash was atypical, being well demarcated, restricted to the antecubital fossae and involving both his penis and scrotum. No other areas were affected. In addition to this we were unable to identify any allergens and he did not have a history of atopy.

## Conclusion

A definitive diagnosis was not reached in this case. However it illustrates the need to revisit the history and examination when the clinical course does not correlate with the expected response and remain open to alternative diagnoses. It also illustrates the need to involve additional specialties particularly when dealing with specialist medication.

## Consent

Written informed consent was obtained from the patient for publication of this case report and accompanying images. A copy of the written consent is available for review by the Editor-in-Chief of this journal.

## Competing interests

The authors declare that they have no competing interests.

## Authors' contributions

TO analysed the case notes, conducted the literature review and drafted the initial manuscript. AN was a major contributor in writing the manuscript. Both authors reviewed the manuscript prior to submission.
